# Supplementary material for: Motivation and Basic Psychological Needs Satisfaction in Active Travel to Different Destinations: A Cluster Analysis with Adolescents Living in Germany
Source: Behav Sci (Basel). 2023 Mar 20;13(3):272. doi: 10.3390/bs13030272 (PMC10045514; doi:10.3390/bs13030272)
Supplement: Supplementary file 1 [file behavsci-13-00272-s001.zip › Supplementary 1.pdf]

Supplementary Table S1. Count, expected count, percentage amount of adolescents traveling active or passive per cluster and adjusted residuals for travel mode to the examined destinations across the identified clusters.

			Cluster						
			1	2	3	4	5	6	total
**Travel mode to school**	passive	Count	57	41	38	43	35	58	272
		Expected Count	42.615	36.828	36.302	41.563	44.193	70.499	272
		% of Cluster	70.4%	58.6%	55.1%	54.4%	41.7%	43.3%	52.6%
		Adjusted Residuals	3.486	1.074	0.440	0.352	-2.195	-2.512	
	active	Count	24	29	31	36	49	76	245
		Expected Count	38.385	33.172	32.698	37.437	39.807	63.501	245
		% of Cluster	29.6%	41.4%	44.9%	45.6%	58.3%	56.7%	47.4%
		Adjusted Residuals	-3.486	-1.074	-0.440	-0.352	2.195	2.512	
	total	Count	81	70	69	79	84	134	517
**Travel mode from school**	passive	Count	54	40	38	45	36	58	271
		Expected Count	42.458	36.692	36.168	41.41	44.031	70.24	271
		% of Cluster	66.7%	57.1%	55.1%	57.0%	42.9%	43.3%	52.4%
		Adjusted Residuals	2.796	0.851	0.474	0.879	-1.917	-2.460	
	active	Count	27	30	31	34	48	76	246
		Expected Count	38,542	33,308	32,832	37,59	39,969	63,76	246
		% of Cluster	33,3%	42,9%	44,9%	43,0%	57,1%	56,7%	47.6%
		Adjusted Residuals	-2,796	-0,851	-0,474	-0,879	1,917	2,460	
	total	Count	81	70	69	79	84	134	517
**Travel mode to friends / relatives**	passive	Count	36	25	21	15	23	19	139
		Expected Count	21.676	18.967	18.696	21.405	22.489	35.766	139
		% of Cluster	45.0%	35.7%	30.4%	19.0%	27.7%	14.4%	27.1%
		Adjusted Residuals	3.922	1.746	0.671	-1.763	0.138	-3.810	
	active	Count	44	45	48	64	60	113	374
		Expected Count	58.324	51.033	50.304	57.595	60.511	96.234	374
		% of Cluster	55.0%	64.3%	69.6%	81.0%	72.3%	85.6%	72.9%
		Adjusted Residuals	-3.922	-1.746	-0.671	1.763	-0.138	3.810	
	total	Count	80	70	69	79	83	132	513
**Travel mode to shopping facilities**	passive	Count	26	26	19	17	17	27	132
		Expected Count	19.575	17.723	17.459	20.369	21.427	35.447	132
		% of Cluster	35.1%	38.8%	28.8%	22.1%	21.0%	20.1%	26.5%
		Adjusted Residuals	1.835	2.464	0.462	-0.946	-1.218	-1.934	
	active	Count	48	41	47	60	64	107	367
		Expected Count	54.425	49.277	48.541	56.631	59.573	98.553	367
		% of Cluster	64.9%	61.2%	71.2%	77.9%	79.0%	79.9%	73.5%
		Adjusted Residuals	-1.835	-2.464	-0.462	0.946	1.218	1.934	
	total	Count	74	67	66	77	81	134	499
**Travel mode to leisure facilities**	passive	Count	37	26	25	18	18	29	153
		Expected Count	23.609	20.543	20.543	23.303	25.142	39.86	153
		% of Cluster	48.1%	38.8%	37.3%	23.7%	22.0%	22.3%	30.7%
		Adjusted Residuals	3.599	1.554	1.269	-1.433	-1.871	-2.402	
	active	Count	40	41	42	58	64	101	346
		Expected Count	53.391	46.457	46.457	52.697	56.858	90.14	346
		% of Cluster	51.9%	61.2%	62.7%	76.3%	78.0%	77.7%	69.3%
		Adjusted Residuals	-3.599	-1.554	-1.269	1.433	1.871	2.402	
	total	Count	77	67	67	76	82	130	499

Note: Table cells highlighted in grey show adjusted residuals that exceed the threshold of +/- 1.96.
