# Supplementary material for: Motivation and Basic Psychological Needs Satisfaction in Active Travel to Different Destinations: A Cluster Analysis with Adolescents Living in Germany
Source: Behav Sci (Basel). 2023 Mar 20;13(3):272. doi: 10.3390/bs13030272 (PMC10045514; doi:10.3390/bs13030272)
Supplement: Supplementary file 1 [file behavsci-13-00272-s001.zip › Supplementary 2.pdf]

Supplementary Table S2. Overview on the amount of adolescents reporting to travel a distance (category) to friends or relatives, shopping facilities or leisure facilities across clusters and mean distance to school per cluster.

		Cluster						
		**1**	**2**	**3**	**4**	**5**	**6**	**total**
**Distance to friends or relatives**	less than 500m	13	10	11	18	10	11	73
	500m-1km	19	11	13	19	17	33	112
	1km-2km	10	15	12	17	25	32	111
	2km-3km	11	6	9	5	10	17	58
	3km-5km	7	9	9	10	4	19	58
	more than 5km	21	19	13	10	17	20	100
	total	81	70	67	79	83	132	512
**Distance to shopping facilities**	less than 500m	11	8	7	14	9	17	66
	500m-1km	29	19	21	22	36	41	168
	1km-2km	12	21	14	20	17	33	117
	2km-3km	5	5	8	11	7	14	50
	3km-5km	7	9	9	2	10	11	48
	more than 5km	15	8	9	10	3	17	62
	total	79	70	68	79	82	133	511
**Distance to leisure facilities**	less than 500m	11	8	7	10	14	15	65
	500m-1km	15	18	11	21	22	27	114
	1km-2km	14	15	16	17	18	25	105
	2km-3km	10	8	9	8	10	21	66
	3km-5km	9	7	11	12	7	19	65
	more than 5km	21	11	11	9	11	22	85
	total	80	67	65	77	82	129	500
**Mean distance to school in km**	*Displayed as mean distance (standard deviation)*	6.78 (0.60)	7.74 (0.78)	6.67 (0.65)	6.85 (0.67)	5.67 (0.54)	6.03 (0.44)	6.54 (5.46)
